# Immune-based therapies in the management of multiple myeloma

**DOI:** 10.1038/s41408-020-00350-x

**Published:** 2020-08-22

**Authors:** Saurabh Zanwar, Bharat Nandakumar, Shaji Kumar

**Affiliations:** 1 Division of Hematology, Mayo Clinic, Rochester, MN USA; 2 Department of Internal Medicine, Mayo Clinic, Rochester, MN USA

**Keywords:** Myeloma, Therapeutics

## Abstract

Multiple myeloma (MM) is a clonal plasma cell malignancy affecting a predominantly elderly population. The continued development of newer therapies with novel mechanisms of action has reshaped the treatment paradigm of this disorder in the last two decades, leading to a significantly improved prognosis. This has in turn resulted in an increasing number of patients in need of therapy for relapsed/refractory disease. Immune-based therapies, including monoclonal antibodies, immune checkpoint inhibitors, and most promisingly, adoptive cellular therapies represent important therapeutic strategies in these patients due to their non-cross resistant mechanisms of actions with the usual frontline therapies comprising of immunomodulatory drugs (IMiDs) and proteasome inhibitors (PIs). The anti-CD38 antibodies daratumumab and more recently isatuximab, with their excellent efficacy and safety profile along with its synergy in combination with IMiDs and PIs, are being increasingly incorporated in the frontline setting. Chimeric antigen receptor–T cell (CART) therapies and bi-specific T-cell engager (BiTE) represent exciting new options that have demonstrated efficacy in heavily pretreated and refractory MM. In this review, we discuss the rationale for use of immune-based therapies in MM and summarize the currently available literature for common antibodies and CAR-T therapies that are utilized in MM.

## Introduction

Multiple myeloma (MM) is a disorder of clonal plasma cells with a median age at diagnosis of around 67 years and accounts for ~10% of all newly diagnosed hematologic malignancies^1^. The incorporation of novel agents into upfront therapy and the introduction of maintenance approaches have led to a sustained improvement in the overall survival (OS) of patients with MM over the past two decades^2^. Unfortunately, MM remains incurable and relapse of the disease is common. The improvement in outcomes of patients with MM has also presented the challenge of treating an increasingly elderly population at relapse, making it important to have drugs with a better efficacy and safety profile to minimize toxicities^3^. A host of new agents have been approved for use in relapsed/refractory MM (RRMM) and potentially the most revolutionary class of therapeutics that have been introduced in the treatment paradigm of MM in the past few years include immune-based therapies targeting the malignant cell clone^4,5^. Immune-based therapy for treatment of MM is not a new concept. Allogenic stem cell transplant has been utilized as a treatment strategy in MM and the presence of a graft versus myeloma effect points toward the efficacy of this approach^6^. The high rates of transplant-related mortality associated with allogenic transplant have made it a less preferred modality, but the potentially curative nature of this treatment makes it unique in the treatment landscape for MM^7^. Immune dysregulation is postulated to be centrally involved in the pathogenesis and disease progression in MM^8^. In this review, we discuss the rationale, targets, and evidence for immune-based therapies in treating MM.

## Immune dysregulation in MM

### Role of immune checkpoints in pathogenesis of MM

A normal cell-mediated immune response is driven by the interaction between antigen presenting cells (APC) and effector T cells, and is orchestrated by the dynamic balance between the activating and inhibitory signaling molecules and cytokines^9^. The B7-CD28 family of proteins, especially cytotoxic tumor lymphocyte antigen 4 (CTLA-4) and programmed cell death-1 (PD-1) are important co-inhibitory molecules that are expressed on T-cells, B-cells, and natural killer (NK) cells^10^. These serve as important immune checkpoints and regulate the production of antigen-specific T-cells thus playing are an important role in maintaining immune tolerance^11^. Programmed cell death-ligand 1 (PD-L1), the ligand for PD-1 receptor, is expressed on APCs and its binding to PD-1 leads to suppression of T-cell activation and immune response. Cytotoxic tumor lymphocyte antigen-4 competes with the co-stimulatory molecule CD28 in binding to CD80 and CD86 thereby limiting the production of antigen-specific T cells^11^. Similarly, the binding of PD-L1 to PD-1 leads to immune inhibition and escape of tumor cells from immune surveillance^11^. Both CTLA-4 and PD-L1 are shown to have increased expression in bone marrow milieu of patients with MM^12,13^.

Another important player involved in the immune regulation of T cells in MM is lymphocyte activation gene-3 (LAG3; CD223). The role of LAG3 as an immune checkpoint was identified in relation to its role in enhancing the function of regulatory T cells (T_regs_) and inhibition of CD8 T-cells^14^. Like PD-1/PD-L1, LAG3 expression can prevent the development of autoimmunity but sustained LAG3 stimulation can be associated with T-cell exhaustion which can potentially contribute to immune escape^10^. Increased expression of LAG3 on T cells in the tumor milieu of MM has been noted in murine models and there is preclinical evidence of synergy between PD-1 and LAG3 inhibition, which could represent an important dual immune targeting strategy^15^. In a small study performed on 16 bone marrow specimens, patients with faster progression of smoldering MM had an expression of negative immune regulatory mediators including LAG3 expression on the T cells in the microenvironment and PD-L1 on the plasma cells^16^. A recent study also demonstrated that increased LAG3 transcript expression in T-cells postautologous transplant was associated with a worse outcome in patients with MM, suggesting potential role of targeting LAG3 in MM^17^. Other important immune checkpoints include T-cell immunoglobulin (TIM)-3 and T-cell immunoreceptor with Ig and ITIM (TIGIT) domains which are currently being studied as potential targets for therapy^18^.

### T_reg_-mediated immune suppression in MM

T_regs_ are involved in the maintenance of self-tolerance and prevention of autoimmunity by exerting an inhibitory effect on immune response through various mechanisms, including release of inhibitory interleukins (IL) like IL-10, TGF-β, and IL-35 that leads to a state of T-cell anergy and immune paresis^19,20^. The bone marrow (BM) and peripheral blood in patients with MM and monoclonal gammopathy of undetermined significance show increased frequency of T_regs_ compared to that in patients of age-matched healthy controls^21^. Also, CTLA-4 is expressed on T_regs_ and plays a role in mediating the immune suppressive effects of T_regs_^13^. The T_regs_ are believed to play an important role in resistance to immune-mediated destruction in MM, making them potentially important drug targets^22^.

### BM microenvironment in immune dysfunction in MM

The BM microenvironment is thought to play a central role in the development and progression of MM^8^. There is an extensive crosstalk between the BM stromal cells (BMSCs), BM endothelial cells and the MM cells which leads to the secretion of cytokines like hepatocyte growth factor (HGF), vascular endothelial growth factor, transforming growth factor-β, stromal cell-derived factor-1α (SDF-1α) among others that promote myeloma cell survival^23^. Additionally, interleukin-6 is released from the interaction of myeloma cells with BMSCs and promotes survival of the malignant cells^24^. The change in cytokine milieu leads to the generation of a Th-17 profile which is associated with an increase in osteoclastogenic activity and MM cell growth^25^. Myeloid-derived suppressor cells (MDSCs) also mediate the suppression of T-cell-mediated immunity against MM cells via interaction with T_regs_ and also play a role in progression and resistance to therapy in MM^26^. Additionally, plasmacytoid dendritic cells (DCs), which are important in their role as APCs are often dysfunctional and release various soluble factors like VEGF, IL-6, SDF-1α among others upon interaction with MM cells, which in turn promote the growth and survival of myeloma cells^27^. Indoleamine 2,3 dioxigenase-1 (IDO-1), an enzyme involved in tryptophan metabolism, is also overexpressed by myeloma cells through the action of HGF, which increases the generation of kynurenines that contribute to the immune suppressive microenvironment in the BM^28^. IDO-1 also leads to the expansion of T_regs_ further contributing to modulation of T-cell function^28^. Thus, various factors contribute to the development of immune dysregulation in MM, which in turn is thought to play a role in promoting growth, prolonging survival, and conferring resistance to therapy of MM cells.

## Immunotherapy in MM: a multifaceted approach

**Fig. 1:**
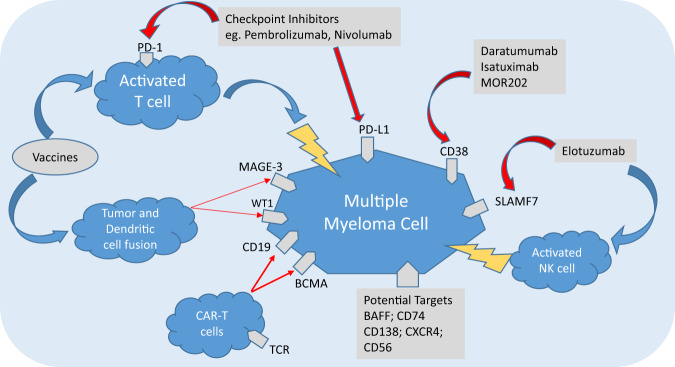
Mechanisms of action of various immunotherapeutic agents for treating multiple myeloma. BAFF B-cell activating factor, BCMA B-cell maturation antigen, CAR-T cells chimeric antigen receptor T cells, CXCR4 chemokine receptor 4, MAGE-3 melanoma-associated antigen-3, NK cell natural killer cell, PD-1 programmed death receptor-1, PD-L1 programmed death receptor ligand-1, SLAMF7 signaling lymphocyte activating molecule family-7, TCR T-cell receptor, WT1 Wilms tumor 1.

Newer agents looking at immune targeting of MM cells can be divided into three broad types (Fig. 1).

1. Monoclonal antibodies (mAbs) targeting antigens expressed by MM cells.
2. Treatment strategies to reverse the immune tolerance towards MM cells—the checkpoint inhibitors.
3. Antitumor activity through the generation of higher and more potent immune effector cells against MM (e.g., genetically engineered T cells, bispecific antibodies, vaccines).

## Targets for mAbs in the treatment of MM

An ideal target antigen for an mAb would be one with a high and uniform expression on the myeloma cells to have sufficient efficacy and a negligible to low expression on other normal cells to avoid off-target side effects. Various antigens expressed on MM cells have been studied and antibodies against many of these have been developed. Of these, CD38, signaling lymphocyte activating molecule family-7 (SLAMF7), and B-cell maturation antigen (BCMA) represent the most important targets and are discussed in detail.

### Physiologic role and clinical efficacy of CD38 targeting

The CD38 antigen is a cell surface glycoprotein that is involved in signal transduction. Engagement of CD38 stimulates intracellular proliferation signals through a cascade involving various signaling molecules like CD31, ZAP-70, and ERK1/2^29^. CD38 also acts as an ectoenzyme that promotes the generation of adenosine through the degradation of nicotinamide adenine dinucleotide. Adenosine in turn is thought to promote survival of myeloma cells via modulation of the immune response toward the malignant clone^30^. As a target, CD38 is uniformly expressed on majority of the myeloma cells^31^. Daratumumab (IgG1κ; fully human), isatuximab (IgG1κ; chimeric), and MOR202 (IgG1λ; fully human) are mAbs developed and tested against CD38. In preclinical studies, all three antibodies have been shown to cause cell death via antibody-dependent cellular cytotoxicity (ADCC) and complement-dependent cytotoxicity, the classic mechanisms in antibody-mediated cytotoxicity^31^. In addition to these, antibody-dependent cellular phagocytosis and apoptosis also play a role in cell kill by these antibodies. Despite the similarities in mechanism of action, there appear to be some differences in these three antibodies and they bind to different epitopes of CD38. Isatuximab appears to be a more potent inducer of ectoenzyme function of CD38 and unlike daratumumab, leads to apoptosis of MM cells without crosslinking of the Fc receptors of the antibody^31^. However, the clinical implication of these differences is not well established presently.

Daratumumab is the CD38 antibody for which the majority of the clinical trial data currently exists. In the phase II SIRIUS trial, daratumumab monotherapy demonstrated an overall response rate (ORR) of 29% in a heavily pretreated population that had received a median of five prior lines of therapy^32^. Lenalidomide has shown to upregulate the expression of CD38 on myeloma cells and immunomodulatory drugs (IMiDs) appear to be natural allies to anti-CD38-directed antibodies^33^. The combination of daratumumab with lenalidomide and dexamethasone (Rd) was found to be highly active with an ORR of 81% and stringent complete response (sCR) rate of 25% in a pretreated population with a median of 2 prior lines of therapy^34^. Recent phase III clinical trials with daratumumab in combination with both proteasome inhibitors (PIs) and IMiDs have demonstrated a remarkable improvement in the rate of deeper responses including higher minimal residual disease negativity, and improved outcomes in patients with MM^35–37^. Some of the important clinical trials of CD38-directed mAbs in the frontline and relapsed setting are highlighted in Table 1.

**Table 1:** Selected clinical studies with CD38- and SLAMF7-directed therapies in multiple myeloma.

Reference and study	Study population	Regimen	Response, (%)	PFS (months)	OS	Grade ≥3 AE (%)
**CD38 antibodies**						
**Daratumumab**						
Mateos et al.^36^ ALCYONE	Newly diagnosed transplant-ineligible multiple myeloma (*n* = 700)	D-VMP vs. VMP	ORR/CR 91/43 vs. 74/24	18-month PFS (%): 72 vs. 50 (HR 0.5; *p* < 0.001)	OS data immature	Anemia/thrombocytopenia /neutropenia/overall (%) 16/34/40 vs. 20/38/39 Grade ≥3 infusion reaction with DARA arm: 4.7%
Facon et al.^101^ MAIA	Newly diagnosed transplant-ineligible multiple myeloma (*n* = 737)	DRd vs. Rd	CR/ MRD neg 48/24 vs. 25/7	Median: NR vs. 31.9 m (HR 0.55; *p* < 0.0001)	OS data immature	Neutropenia/anemia infections 50/12/32 vs. 35/20/23
Moreau et al.^102^ CASSIOPEIA	Newly diagnosed transplant eligible multiple myeloma (*n* = 1085)	D-VTd vs. VTd	sCR/MRD neg (post ASCT) 29/64 vs. 20/44	18-month PFS (%) 93 vs. 85 (HR 0.43; *p* < 0.001)	OS data immature	Neutropenia/thrombocytopenia/peripheral neuropathy 28/11/9 vs. 15/7/9
Voorhees et al.^103^ GRIFFIN	Newly diagnosed transplant eligible multiple myeloma (*n* = 207)	D-RVd vs. RVd	sCR(post ASCT)/ MRD neg 42/51 vs. 34/20	24-month PFS (%) 96 vs. 90 (HR-NA)	OS data immature	Neutropenia/anemia/thrombocytopenia 41/9/16 vs. 22/6/9 Grade ≥3 infusion reaction with DARA arm: 6.1%
Dimopoulos et al.^35^ POLLUX	≥1 lines of therapy with response and progression; not refractory or intolerant to lenalidomide (*n* = 569)	DRd vs. Rd	ORR/CR: 93/43 vs. 76/19	18-month PFS (%): 78 vs. 52 (HR 0.37; *p* < 0.001)	12-month OS (%): 92 vs. 87	Neutropenia/anemia/thrombocytopenia (%) 31/12/13 vs. 37/20/13 Grade ≥3 infusion reaction with DARA arm: 5.6%
Palumbo et al.^37^ CASTOR	≥1 lines of therapy with response and progression; not refractory or intolerant to bortezomib (*n* = 498)	DVd vs. Vd	ORR/CR: 83/19 vs. 63/9 (*p* < 0.001)	12-month PFS (%): 61 vs. 27 (HR 0.39; *p* < 0.001)	OS data immature	Anemia/thrombocytopenia/neutropenia/overall 14/45/13/76 vs. 16/33/4/62 All grade infusion reaction in DARA arm 45.6%
Usmani et al.^104^ CANDOR	1–3 lines of prior therapy, with ≥PR to at least 1 prior line (*n* = 466)	KdD vs. Kd	CR/MRD neg at 12 m 29/12.5 vs. 10/1.3	Median: NR vs. 15.8 m (HR 0.63; *p* < 0.01)	OS data immature	Overall grade ≥3 AE 82 vs. 74
Usmani et al.^105^ (SIRIUS and GEN501 combined)	≥2–3 lines of therapy including PI and IMiD (*n* = 148)	DARA	ORR/CR 31/5	Median: 4 months	Median OS: 20.7 months	Anemia/thrombocytopenia/neutropenia 18/9/8 Grade ≥3 infusion reaction with DARA: 2.7%
**Isatuximab**						
Attal et al.^106^ ICARIA-MM	≥2 prior lines including IMid and PI (*n* = 307)	IPd vs. Pd	ORR/CR 93/7 vs. 54/2	Median: 11.5 vs. 6.5 (HR 0.6; *p* = 0.001)	OS data immature	Overall grade ≥3 AE 83 vs. 47 Grade ≥3 infusion reaction with isatuximab-2%
Moreau et al.^107^ IKEMA	1–3 lines of prior therapy, no prior carfilzomib (*n* = 302)	IKd vs. Kd	ORR/CR 87/40 vs. 83/28	Median: NR vs. 19.1 m (HR 0.53; *p* < 0.001)	OS data immature	Anemia/thrombocytopenia/neutropenia/overall 22/30/19/76 vs. 20/24/7/67 Grade ≥3 infusion reaction with isatuximab-0.6%
**SLAMF7 antibody**						
Lonial et al.^42^ ELOQUENT-2	≥1 lines of therapy, not refractory/ intolerant to lenalidomide (*n* = 646)	ERd vs. Rd	ORR/CR: 79/4 vs. 66/7	Median PFS 19.4 vs. 14.9 (HR 0.7; *p* < 0.001)	Median OS NR	Anemia/thrombocytopenia/neutropenia (%) 19/19/34 vs. 21/20/44
Jakubowiak et al.^108^	1–3 lines of prior therapy, not refractory or intolerant to PI (*n* = 152)	EVd vs. Vd	ORR/CR: 66/4 vs. 63/3	Median PFS: 9.6 vs. 6.9 (HR 0.72; *p* = 0.09)	1-year OS (%): 85 vs. 74 (HR: 0.61; p-NA)	Infections/thrombocytopenia/diarrhea/overall (%) 21/9/8/71 vs. 13/17/4/60
Dimopoulos et al.^43^ ELOQUENT-3	≥2 lines of therapy, relapsed/ refractory to lenalidomide and a PI (*n* = 117)	EPd vs. Pd	ORR/CR 53/5 vs. 26/2	Median PFS 10.3 vs. 4.7 (HR 0.54; *p* = 0.008)	Median OS NR	Anemia/thrombocytopenia/neutropenia (%) 10/8/13 vs. 20/5/27

*AE* adverse effects, *ASCT* autologous stem cell transplantation, *CI* confidence interval, *CR* complete response, *DARA* daratumumab monotherapy, *DOR* duration of response, *DRd* daratumumab, lenalidomide, dexamethasone, *D-RVd* daratumumab, lenalidomide, bortezomib, dexamethasone, *DVd* daratumumab, bortezomib, dexamethasone, *D-VMP* daratumumab, bortezomib, melphalan, prednisolone, *D-VTd* datarumumab, bortezomib, thalidomide, dexamethasone, *ERd* elotuzumab, lenalidomide, dexamethasone, *EPd* elotuzumab, pomalidomide, dexamethasone, *EVd* elotuzumab, bortezomib, dexamethasone, *IMiD* immunomodulatory drugs, *IKd* isatuximab, carfilzomib, dexamethasone, *IPd* isatuximab, pomalidomide, dexamethasone, *Kd* carfilzomib, dexamethasone, *KdD* carfilzmib, dexamethasone, daratumumab, *HR* hazard ratio, *m* months, *MRD neg* minimal residual disease negative, *NA* not available, *NR* not reached, *ORR* overall response rate, *OS* overall survival, *Pd* pomalidomide, dexamethasone, *PFS* progression-free survival, *PI* proteasome inhibitor, *Rd* lenalidomide, dexamethasone, *RVd* lenalidomide, bortezomib, dexamethasone, *SAE* serious adverse event, *sCR* stringent complete response, *Vd* bortezomib–dexamethasone, *VMP* bortezomib, melphalan, prednisolone, *VTd* bortezomib, thalidomide, dexamethasone.

### Signaling lymphocyte activating molecule family-7 (SLAMF7)

This is a cell surface molecule expressed on MM cells as well as on other lymphocytes, including NK cells and functions to modulate the normal immune response and has been shown to promote survival function of myeloma cells^38^. Elotuzumab (E) is a fully humanized mAb-directed against SLAMF7. The Fab fragment of elotuzumab binds to the extracellular domain of SLAMF7 and the Fc portion attaches to the CD16 receptor of NK cells^39^. Attachment of elotuzumab to NK cells leads to their activation and degranulation, ultimately causing myeloma cell death by ADCC^40^. In clinical trials, elotuzumab monotherapy has demonstrated only modest activity, with few patients achieving disease stabilization as the best response^41^. The phase III ELOQUENT-2 trial compared ERd and Rd in 321 patients with previously treated MM (at least 1 prior line of therapy) that were not refractory to lenalidomide^42^. The ORR was significantly better for the elotuzumab arm (79% vs. 66%; *p* < 0.01). At a median follow-up of 24.5 months, the median PFS was 19.4 months for ERd versus 14.9 months for Rd (hazard ratio [HR] 0.7; 95% confidence interval [CI] 0.57–0.85; *p* < 0.01)^42^. Elotuzumab has also been studied in combination with pomalidomide and dexamethasone (EPd) in a phase 2 study (ELOQUENT-3) for RRMM with at least two prior lines of therapy, including lenalidomide and PI. The primary endpoint of the study was PFS and EPd was associated with a significant improvement in PFS (10.4 vs. 4.7 months; HR 0.54, *p* = 0.008) compared to pomalidomide and dexamethasone (Table 1)^43,44^. Elotuzumab in combination with lenalidomide, bortezomib, and dexamethasone (RVd) has also been studied by Usmani et al. in a phase 1 study of eight patients with newly diagnosed MM and demonstrated a good safety profile with a comparable toxicity profile to RVd^45^.

### B-cell maturation antigen (BCMA)

A critical part of immune-based therapy is the selection of an appropriate tumor-specific antigen, which should ideally be uniformly expressed on the MM plasma cells with limited or no expression on the normal cells^46^. This can be challenging, especially due to the development of sub-clones that can impart potential phenotypic differences to the MM cells^46^. A well-suited target appears to be BCMA (CD269), which is a member of the tumor necrosis factor receptor family^47^. Its expression is restricted to some B-cell lineage cells with fairly uniform expression on normal plasma cells and MM cells with almost no expression on non-hematologic cells, making it an ideal target antigen^48^. BCMA-directed therapy represents an important treatment option in heavily pretreated MM^49,50^. Belantamab mafodotin (GSK2857916) is an antibody drug conjugate (ADC) consisting of an afucosylated anti-BMCA IgG1 mAb conjugated to monomethyl auristin-F (MMAF). The ADC acts by binding to the surface of MM cells expressing BCMA followed by internalization of MMAF which acts by microtubule disruption and inhibition of mitosis. The afucosylated arm also helps to promote ADCC against BCMA expressing cells^51^. Belantamab mafodotin has been evaluated in RRMM who had received prior alkylator therapy, PIs, IMiDs, and stem cell transplant and demonstrated promising activity in phase 1 study with a 60% ORR in the 3.4 mg/kg cohort, including one sCR and two complete responses (CR). The median PFS was 7.9 months (95% CI: 3.1-not reached)^51^. Corneal side effects (blurred vision, dry eye, and photophobia) were common adverse events noted in 63% of the patients in the 3.4 mg/kg dose cohort. These corneal side effects were reversible and attributed to the payload (MMAF) used in belantamab mafodotin with similar adverse effects have been consistently described in various other ADCs incorporating MMAF^52^. Grade ≥3 thrombocytopenia and anemia were noted in 34% and 14% patients, respectively^51^. The subsequent phase 2 trial (DREAMM-2) studied belandatamab mafodotin in two dose regimens of 2.5 and 3.4 mg/kg^53^. The median lines of prior therapies were 6 and 7, respectively. Majority of the patients in both the cohorts were refractory to PIs (76% and 75% refractory to bortezomib, 65% and 58% refractory to carfilzomib), IMiDs (90% and 89% refractory to lenalidomide, 87% and 78% refractory to pomalidomide), and daratumumab (100% and 92%) in the 2.5 and 3.4 mg/kg dose arms, respectively^53^. At the data cutoff date, the ORR was 31% (30/97) and 34% (34/99) in the 2.5 and 3.4 mg/kg dose arms, respectively. Grade ≥3 keratopathy was noted in 27% and 21% in the 2.5 and 3.4 mg/kg dose arms, respectively, and was the most common reason for permanent discontinuation of therapy. After a median follow-up of 6.5 months, the median PFS was 2.9 months (95% CI: 2.1–3.7) in the 2.5 mg/kg cohort and 4.9 months (95% CI: 2.3–6.2) in the 3.4 mg/kg cohort. The OS data were not mature at the time of reporting^53^. Newer ADCs utilizing BCMA as a target, including MEDI2228 (incorporating Pyrrolobenzodiazepine) and HDP-101 (incorporating Amanitin), are also under evaluation^50^.

### Antibodies against other targets

Antibodies have also been developed against other promising antigens in MM. Tabalumab, an mAb against B-cell activating factor (BAFF), has been tested in a dose-escalation phase I study in combination Vd in RRMM. The combination was well tolerated with an ORR of 56%^54^. However, a phase II study of tabalumab 100 or 300 mg in combination with Vd failed to show an improvement in the PFS of patients with tabalumab and Vd compared to the placebo-Vd arm^55^. Milatuzumab is a humanized anti-CD74 antibody that has demonstrated disease stabilization in a heavily pretreated population of MM when used as monotherapy but objective responses were lacking^56^. Indatuximab ravtansine, an ADC comprising of an mAb against CD138 conjugated with maytansinoid DM4 (microtubule-binding cytotoxic agent), demonstrated only modest clinical activity as monotherapy in RRMM, but was found to be fairly well tolerated in combination with dexamethasone and lenalidomide or pomalidomide, with a median duration of response of 21 months and ORR of 77% in evaluable patients^57^. Antibodies against many other targets, including IL-6 and CXCR4, have also been developed and are in early phases of testing (Table 2)^58^.

**Table 2:** Additional monoclonal antibodies being studied in the treatment of MM.

	Target	Phase of study	Response
Monoclonal antibody			
Tabalumab^54^	BAFF	II	ORR of 56% in RRMM in combination with Vd; a phase II combination therapy study failed to show improvement in progression-free survival compared to placebo
Milatuzumab^56^	CD74	I/II	No OR as monotherapy in RRMM; 26% had SD for >3 months (median 5 lines of therapy)
Siltuximab^109^	IL-6	II	No OR as monotherapy; combination with dexamethasone showed 17% PR (median 4 lines of prior therapy)
**Antibody drug conjugates**			
Indatuximab ravtansine^57^	CD138 (payload: maytansinoid DM4)	I/IIa	ORR of 78% with Rd in RRMM (median 4 lines of prior therapy)
Belanatmab mafadotin (GSK2857916)^53^	BCMA (payload: MMAF)	II	ORR: 31% (30/97) in 2.5 mg/kg dose and 34% (34/99) in the 3.4 mg/kg dose in a heavily pretreated and refractory population
**Antibodies against immune checkpoints**			
Pembrolizumab^65^	PD-L1	I	ORR of 76% in combination with Rd (median 3 lines of prior therapies)
Nivolumab^61,66^	PD-1	I	Best response of SD in 63% as monotherapy in RRMM (*n* = 27) with OR in 1 (4%) patient; no objective response noted with combination of nivolumab with ipilimumab in the subset of RRMM (*n* = 7)
Atezolizumab^67^	PD-L1	Ib	VGPR or better with atezolizumab combination therapy: 67% (4/6) in combination with daratumumab and pomalidomide 44% (3/7) with lenalidomide and daratumumab 50% (3/6) with daratumumab (1–3 prior lines of therapy)

*BAFF* B-cell activating factor, *BCMA* B-cell maturation antigen, *MMAF* monomethyl auristatin F, *OR* objective response, *ORR* overall response rate, *PR* partial response, *Rd* lenalidomide–dexamethasone, *RRMM* relapsed refractory multiple myeloma, *SD* stable disease, *Vd* bortezomib–dexamethasone.

## Strategies to reverse the immune tolerance towards MM cells: PD-1/PD-L1-directed therapy in MM

The PD-1/PD-L1 axis is a negative co-stimulatory pathway that plays a crucial role in regulating immune response. While physiologically it is important to prevent autoimmunity, its overexpression leads to immune evasion and development of tolerance against tumor cells in various malignancies^10^. Plasma cells in patients with MM have demonstrated to have increased PD-L1 expression^12^. Similarly, the circulating T cells and NK cells in patients with MM demonstrate increased expression of PD-1 receptor^59^. The binding of PD-L1 on myeloma cells to the PD-1 receptor on NK cells and T cells leads to a decline in the Th-1 cytokines resulting in T-cell apoptosis and attenuation of T-cell immune effector functions^60^. Despite a strong rationale for the role of immune checkpoint inhibitors in MM, no objective response was noted in the phase I study of pembrolizumab (PD-L1 antibody) monotherapy for RRMM, with the best response achieved being disease stabilization^61^. In a phase I study looking at efficacy of nivolumab in hematologic malignancies, the best response with nivolumab monotherapy in the subset of patients with RRMM (*n* = 27) was stable disease (63%) with objective response noted in only one patient (4%)^61^.

Lenalidomide and pomalidomide appear to enhance sensitivity of checkpoint inhibitors in MM and a combination of these regimens can inhibit the proliferative effects of the BMSCs on myeloma cells and reverse the immunoparesis due to the MDSCs^62^. In a phase I dose-escalation study, the combination of lenalidomide and dexamethasone with the PD-L1 antibody pembrolizumab demonstrated an ORR of 76% with responses noted in patients that were refractory to lenalidomide as well^63^. Pembrolizumab has also been studied in combination with pomalidomide and dexamethasone (KEYNOTE-183) in a predominantly PI and lenalidomide refractory population^64^. The combination demonstrated an ORR of 60 % in the double-refractory subset; the common grade ≥3 hematologic toxicities included neutropenia (40%) and anemia (21%), whereas interstitial pneumonitis was noted in 15% patients and was predominantly low grade^64^. The KEYNOTE-185 is a phase III study comparing pembrolizumab+Rd and Rd in patients with newly diagnosed transplant-ineligible MM^65^. Preliminary data after recruitment of 301 patients out of the target of 640 patients suggest higher rates of toxicity, especially immune-mediated toxicities (including hyper/hypothyroidism, colitis, skin reactions) in the pembrolizumab arm^65^. In view of these adverse signals, the FDA had put the KEYNOTE-183 and KEYNOTE-185 studies on hold at the time of this write-up.

Nivolumab was also studied in combination with ipilimumab but the combination did not demonstrate any objective response in the subset of RRMM (*n* = 5)^66^. A phase 1b clinical trial presented its abstract form (NCT02431208) studied atezolizumab in combination with daratumumab (*n* = 11) with or without addition of lenalidomide (*n* = 7) or pomalidomide (*n* = 6)^67^. A very good partial response (VGPR) or better was observed in 50% (3/6) patients treated with atezolizumab with daratumumab with 1–3 prior lines of therapy, 43% (3/7) patients treated with atezolizumab, daratumumab, lenalidomide and 67% (4/6) patients treated with atezolizumab, daratumumab, pomalidomide. The sample size was small to draw reliable conclusions and were no new safety signals reported^67^. Concerns seen in the KEYNOTE-183 and KEYNOTE-185 studies also resulted in complete or partial hold on vast majority of clinical trials of combinations of various checkpoint inhibitors (nivolumab, durvalumab, atezolizumab, etc.) with IMiDs at the time of this write-up. Phase II study of nivolumab and lenalidomide (NCT03333746) in RRMM and phase I study of nivolumab-pomalidomide-dexamethasone±elotuzumab (NCT03023527) in RRMM have been terminated. Similarly, studies of nivolumab combinations (NCT02903381) and atezolizumab monotherapy (NCT02784483) in asymptomatic/smoldering MM have been either suspended or terminated. The most up to date details of the study status of PD-1/PD-L1 inhibitors in MM can be found at www.clinicaltrials.gov.

## Enhancing the generation of myeloma-specific T cells and immune effector cells

### Chimeric antigen receptor-T cell (CAR-T) therapy

CAR-T cell therapy is a form of adoptive cellular therapy wherein the host’s T cells are genetically engineered to express tumor-specific antigens. Chimeric antigen receptors consist of antigen recognition domains and T-cell signaling moieties that are expressed on T-cells cultured from the patient through the use of vectors^68^. CAR-T cells can selectively target the antigens based on the antigen recognition domains expressed on these cells, thus imparting specificity to the T-cell responses resulting in a more effective tumor cell kill with decreased off-target effects, all without any human leukocyte antigen restriction^69^. The first-generation CAR-T cell therapies lacked a co-stimulatory domain and were associated with only modest responses^70^. However, the second-generation CAR-T cell therapies incorporated co-stimulatory domains, commonly CD28 or 4–1BB, and have shown substantial improvement in the efficacy of these CAR-T cells^71^. The generation of CAR-T cells is followed by administration of a conditioning chemotherapy to the patient, typically cyclophosphamide or combination of cyclophosphamide with fludarabine, followed by reinfusion of the CAR-T cells into the patient^69^.

The first-in-human clinical trial with BCMA-directed CAR-T cells in a heavily pretreated population (median of seven prior lines of therapy) demonstrated that the efficacy and toxicity of CAR-T therapy were dose dependent^72^. In the two patients treated at the highest dose of CAR-T (9 × 10^6^ CAR-T cells/kg), one patient achieved an sCR lasting for 17 weeks and another patient achieved a VGPR 28 weeks after the infusion of CAR-T cells; the responses were lower in patients treated at lower doses, with only one of the ten patients going on to achieve a VGPR^72^. The toxicity profile was similar to that seen with CAR-T cell therapy in patients with acute leukemia, with cytokine release syndrome (CRS) being the most noticeable unique toxicity^72^. CRS is a unique toxicity noted with therapies acting via T-cell proliferation strategies (CAR-T and bispecific antibodies) and represents a systemic inflammatory response that can present with varied clinical manifestations including fever, fatigue, arthralgias, rash, and hypotension. The initial lack of awareness regarding the manifestations of CRS led to unfavorable outcomes including mortalities in early clinical trials with CAR-T therapy. Subsequently, the improved understanding of CRS and increased vigilance for the same has helped in earlier institution of treatment with corticosteroids and the anti-IL6 antibody tocilizumab for CRS^73^. Neurological toxicity is also common with CAR-T therapy and can present with a broad variety of symptoms ranging from confusion to the more severe ones in the form of aphasia and encephalopathy^74^.

Idecabtagene vicleucel (ide-cel, bb2121) is a second-generation BCMA-directed CAR-T cell therapy that incorporates 4–1BB as a co-stimulatory domain and has shown promising results^75^. The phase I dose-escalation study included patients with heavily pretreated MM patients with ≥50% BCMA expression and a measurable disease. The generation of the CAR-T cells was successful in all patients. The patients received 50 × 10^6^, 150 × 10^6^, 450 × 10^6^, or 800 × 10^6^ CAR-T cells in the dose-escalation phase and 150 × 10^6^–450 × 10^6^ in the expansion phase of the study^75^. In the 33 evaluable patients, the ORR was 85% with 15 (45%) patients achieving a CR. Notably, the ORR was an impressive 74% in patients with high-risk cytogenetics in this trial. The rate of CRS was 76% with grade ≥3 CRS seen in 6% patients. Neurologic toxicities were noted on 42% patients, with a vast majority being grade 1 or 2. After a median follow-up of 11 months, the median PFS with ide-cel was an impressive 11.8 months (95% CI: 6.2–17.8 months)^75^. Early phase 2 results of ide-cel (KarMMa) were recently presented at the American Society of Clinical Oncology 2020 meeting^76^. The majority of the included patients were refractory to PIs, IMiDs, and CD38-directed therapy. Of the 140 patients that had undergone leukapheresis, 128 had received ide-cel infusion at the time of reporting. The success rate for manufacturing of ide-cel was 99% and patients were treated in doses of 150 × 106 (*n* = 4), 300 × 106 (*n* = 70), and 450 × 106 (*n* = 56). Peak CAR-T population was noted at day 11 of infusion and the ORR and CR for the entire cohort was 73% and 33%, respectively which met the primary endpoint of the study. Higher response rates were noted with escalating doses (ORR/CR of 50%/25%, 69%/29%, and 82%/39% in 150 × 106, 300 × 106, and 450 × 106 dose cohorts, respectively)^76^. The efficacy was maintained across all major subgroups. After a median follow-up of 13.3 months, the median PFS also demonstrated a target dose-based increment with median PFS of 2.8, 5.8, and 12.1 months at dose of 150 × 106, 300 × 106, and 450 × 106, respectively. The PFS improved with the depth of response with a median PFS of 20 months in patients achieving ≥CR. The OS data for ide-cel was not mature at the time of the presentation. The rate of CRS was higher with increased target dose (50%, 76%, and 96% in 150 × 106, 300 × 106, and 450 × 106 target dose cohorts, respectively). Overall, CRS was predominantly low grade with only 6% patients developing grade ≥3 CRS (one patient had grade 5 CRS)^76^.

Phase I study results for another BCMA-directed CAR-T therapy with 4–1BB co-stimulatory signaling domain have been reported recently^77^. There was no pre-specified BCMA expression level required for inclusion in the study. The 25 patients included in the study were treated in three cohorts: BCMA CAR-T cells alone at a dose of 1–5 × 108 (cohort 1), 1.5 g/m^2^ of cyclophosphamide with 1–5 × 107 BCMA CAR-T cells (cohort 2), and the third group with 1.5 g/m^2^ of cyclophosphamide with 1–5 × 108 BCMA CAR-T cells (cohort 3). The response rates with therapy were 44% in cohort 1, 20% in cohort 2 and 64% in cohort 3. The rate of CRS was 88% with grade 3–4 CRS noted in 32% patients. Grade 3–4 neurological toxicity was noted in three (12%) patients^77^. Another promising CAR-T construct is JNJ-4528 that targets CD3 and two epitopes of BCMA. Efficacy and safety profile appears to be excellent and updated results of the CARTITUDE-1 study demonstrate an ORR of 100% with 86% achieving an sCR^78^. Orvacabtagene autoleucel (orva-cel) is another promising BCMA-directed CAR-T therapy with a low affinity for soluble BCMA that has demonstrated an excellent manufacturing success rate (100% at the time of reporting) with low rates of grade ≥3 CRS (3% for the entire cohort) and excellent efficacy profile with CR/sCR rates of 36% for the entire cohort in the recently reported results of the phase 1/2 EVOLVE study^79^. The BCMA-directed CAR-T cell therapy appears to be a very promising treatment strategy as we await further clinical trial data with a longer follow-up. Important CAR-T therapy studies are summarized in Table 3. Development of CAR-T therapy against CD38 and CD138, both expressed uniformly on MM cells, has been successful in preclinical setting and likely to add to this rapidly expanding field^80,81^.

**Table 3:** Summary of important clinical trials incorporating generation of myeloma-specific T cells and immune effector cells.

Reference (study sample size)	Target	Co-stimulatory domain/construct	Lympho-depletion Regimen	Response and outcomes	Cytokine release syndrome
**Chimeric antigen receptor T cell (CAR-T) therapies**					
Raje et al.^75^ (Ide-cel) Phase 1 (*n* = 33)	BCMA	4–1BB co-stimulatory domain (bb2121)	Fludarabine 30 mg/m^2^ + cyclophosphamide 300 mg/m^2^ (on day −5, −4, and −3)	ORR: 85%; sCr: 36%; dose-dependent effect with no VGPR or better below a dose of 150 × 106; median PFS 11.2 months	76% (*n* = 25); grade ≥3 in 6% (*n* = 2)
Munshi et al.^76^ (Ide-cel) Phase 2 (*n* = 140)	BCMA	4–1BB co-stimulatory domain (bb2121)	Fludarabine 30 mg/m^2^ + cyclophosphamide 300 mg/m^2^ (on day −5, −4, and −3)	ORR: 73%, CR = 33%, dose-dependent effect; median PFS 12.1 months at 450 × 106 target dose	84% (*n* = 107); grade ≥3 in 6% (*n* = 6)
Cohen et al.^77^ (*n* = 25)	BCMA	4–1BB co-stimulatory domain	Cyclophosphamide 1.5 g/m^2^ on day −3 (in two out of the three study cohorts)	ORR entire study: 48% (12/25); ORR improved to 55% (11/20) in patient with dose of 1 × 10^8^–5 × 10^8^ CART-BCMA cells; median PFS _῀_2 months	88% (*n* = 22); grade ≥3 in 32% (*n* = 8)
Brudno et al.^110^ (*n* = 16)	BCMA	CD28 co-stimulatory domain	Fludarabine 30 mg/m^2^ + cyclophosphamide 300 mg/m^2^ (on day −5, −4, and −3)	ORR: 81%; ≥VGPR: 63% median EFS: 7.8 months	94% (*n* = 15); grade ≥3 in 38% (*n* = 6)
Zhao et al.^111^ (*n* = 57)	BCMA (targeting 2 BCMA epitopes)	4–1BB co-stimulatory molecule (LCAR-B38M)	Cyclophosphamide 300 mg/m^2^ (on day −5, −4, and −3)	ORR: 88% (50/57) CR: 68% (39/57) median PFS: 15 months (at median follow-up of 8 months)	90% (*n* = 51); grade ≥3 in 7% (*n* = 4)
Xu et al.^112^ (*n* = 17)	BCMA (targeting 2 BCMA epitopes)	4–1BB co-stimulatory molecule (LCAR-B38M)	Cyclophosphamide 300 mg/m^2^ (on day −5, −4, and −3) +/−Fludarabine	ORR: 88% (15/17) sCR: 76% (13/17) PFS rate: 53% at 12 months	100% (*n* = 17); grade ≥3 in 41% (*n* = 7), including 1 death
Berdeja et al.^78^ (CARTITUDE-1; *n* = 29)	BCMA (targeting 2 BCMA epitopes)	4–1BB constimulatory molecule (JNJ-4528)	Fludarabine 30 mg/m^2^ + cyclophosphamide 300 mg/m^2^ (on day −5, −4, and −3)	ORR: 100% (*n* = 29) sCR: 86% (25/29)	93% (*n* = 27); grade ≥3 in 7% (*n* = 2)
Mailankody et al.^79^ (EVOLVE; orva-cel; *n* = 62)	BCMA	4–1BB constimulatory molecule	Fludarabine 30 mg/m^2^ + cyclophosphamide 300 mg/m^2^ (on day −5, −4, and −3)	Entire cohort (*n* = 62): ORR: 92% sCR/CR; 36%	89% (*n* = 55); grade ≥3 in 3% (*n* = 2)
**Bispecific monoclonal antibodies**					
Topp et al.^90^ (*n* = 42)	BCMA	Bispecific antibody against BCMA and CD3	NA	ORR: 70% (7/10) at the dose of 400 µg/d sCR: MRD negative 5/10 (400 µg/d)	Grade 2–3 CRS in 3 patients
Costa et al.^91^ (*n* = 19)	BCMA	Bivalent BCMA target with monovalent CD3 target	NA	ORR: 83% (10/12) at ≥6 mg dose; MRD negative in 9/10 patients	All grade CRS 89% (17/19); 94% (16/17) were grade 1–2
Usmani et al.^92^ (Teclistamab; JNJ- 64007957) (*n* = 66)	BCMA	Bispecific antibody against BCMA and CD3	NA	ORR: 78% (7/9) at the highest dose; MRD negative in 2/2 evaluated patients	All grade CRS 56% (37/66); all CRS events grade 1–2
**Vaccine therapy**					
Rosenblatt et al.^98^ (*n* = 36)	Myeloma cells (tumor-specific immunity)	Myeloma cells fused with autologous dendritic cells	Postautologous transplant setting	Deeping of response in 24% (from PR post ASCT to Cr or nCR at 3 months); ≥VGPR: 78%	NA

*BCMA* B-cell maturation antigen, *CRS* cytokine release syndrome, *Cy* cyclophosphamide, *EFS* event free survival, *Ide-cel* idecabtagene vicleucel (bb2121), *MRD* minimal residual disease, *NA* not applicable, *ORR* objective response rate, *orva-cel* orvacabtagene autoleucel, *PFS* progression-free survival, *sCR* stringent complete response.

There are some hindrances with the use of CAR-T cell therapy in MM. The cells may lose the tumor-specific antigen over time leading to loss of response as was seen in first-generation CARs^46^. The incorporation of a second co-stimulatory molecule in the structure of the CAR-T cells, in addition to the CD3ζ T-cell activation domain, helps to generate a more effective and long-lasting immune response as has been seen in the more recent clinical trials^76,82^. Nonetheless, most patients still relapse after CAR-T therapy. Also, the tumor microenvironment in MM is immune suppressive and may hamper an adequate T-cell response after infusion of these cells^8,23^. Further alterations in the CAR structure like targeting multiple antigens to account for multiple clones may help to overcome some of the limitations^83^.

### Bispecific monoclonal antibodies (BsMAb)

A bispecific monoclonal antibody (BsMAb) concomitantly binds to two different antigens, commonly one being on T cells and the other being a tumor-associated antigen, thereby redirecting the cytotoxic T cells toward the tumor cells. The BsMAb therapy is similar in its mechanism to CAR-T therapy in the sense that both rely on host’s T cells to elicit antitumor response. However, BsMAb comes with the obvious advantage of not requiring a processing period that is associated with the generation of CAR-T cells and hence have earned the appellation of off-the-shelf CAR-T therapy^84^. Various bispecific antibody platforms are currently in clinical trials. The BiTE^®^ (bispecific T-cell engager; Amgen, Thousand Oaks, CA, USA) platform for BsMAb consists of two single-chain variable fragments (scFv), one binding to CD3 on T cells and the other to a tumor-associated antigen^85^. DuoBody^®^ (Genmab A/S, Copenhagen, Denmark) is another bispecific antibody platform that consists of heavy and light chain homodimers from two different antibodies that fused together with a controlled Fab-arm exchange at matched point CH-3 mutations to generate a single monoclonal IgG1 heterodimer. The potential advantage of this platform is that it is stable and retains the functional and structural integrity of native IgG molecule^86,87^. Another platform in clinical trials is Dual-Affinity Re-Targeting (DART^®^, Macrogenics Inc.) which incorporates c-peptide disulfide linkages to further stabilize the two different diabodies^88^. Currently, the majority of clinical trial data for BsMAbs in MM is limited to the BiTE^®^ platform.

There is good preclinical evidence of selective lysis of BCMA positive myeloma cells in ex vivo assays by bispecific antibody targeting BCMA/CD3^89^. A first-in-human study of the BCMA-directed BiTE^®^ AMG420 comprised of 6-week cycles of the experimental drug given for 5 or less cycles depending upon toxicity and progression. Of the 42 heavily pretreated patients enrolled in the trial, 13 (31%) had an objective response. At the tolerated dose of 400 µg/day dose, seven out of ten patients responded. More importantly, five out of these seven patients achieved an sCR^90^. Three patients developed grade 2–3 CRS and treatment-related adverse effect associated mortality was noted in two patients (adenoviral infection associated fulminant hepatitis and pulmonary aspergillosis)^90^.

Another newer BsMAb is CC-93269 which binds bivalently to BCMA and monovalently to CD3ε in a 2:1 fashion^91^. In a phase I study of 19 patients with RRMM with ≥3 previous lines of therapy (BCMA-therapy naive), CC-93269 was studied in doses ranging from 0.15 to 10 mg^91^. All patients were heavily pretreated (median 6 lines of therapy) with a vast majority of patients being refractory to daratumumab as well as last PI and/or IMiD. At doses ≥6 mg in cycle 1, CC-93269 demonstrated remarkable activity with 10 out of 12 (83%) patients achieving a ≥PR and 58% achieving ≥VGPR. In the ten patients that underwent MRD testing, nine of these achieved MRD negativity^91^. The toxicity profile was noticeable for CRS in 17 out of the 19 patients (89%), which was predominantly grade 1–2 (in 16/17 patients with CRS), while one of the 17 patients being treated with 10 mg on day 8 died in the setting of CRS. Cytopenias and infections represented the other common grade ≥3 adverse effects. Data for PFS and OS were not mature at the time of this write-up^91^.

A recently reported phase 1 study of Teclistamab (JNJ-64007957), a bispecific BCMA/CD3 antibody, demonstrated a good tolerability and safety profile with 7/9 (78%) patients achieving a response at the highest dose^92^. Another BCMA/Cd3 bispecific antibody under initial phases of in-human testing is REGN5458. Preliminary results of three patients treated at a starting dose of 3 mg revealed responses in two patients with disease progression the third patient at first assessment. No dose-limiting toxicities were noted and additional data are awaited^93^.

### Vaccine therapy for MM

Strategies for developing vaccines against MM have been around for more than two decades now. Most of the initial strategies involved noncellular approaches employing tumor antigens for inducing immune response. The most commonly employed antigen was the idiotypic antigen that forms the variable fragment of the monoclonal immunoglobulin in MM^5^. Idiotypic antigen-based vaccines had demonstrated poor immunogenicity and have been supplanted by more specific tumor antigens like Cancer Testis Antigens MAGE-3 and NY-ESO-1, WT1, and CS1 amongst others^94^. Tumor cell lysates and apoptotic tumor cells have also been used as the source of antigen for the generation of vaccines in order to achieve better immunogenicity, but clinical benefit with these strategies remained elusive^95^.

A promising approach appears to be the use of fusion vaccines that employ autologous DCs fused with tumor cells. DCs are potent APCs and this fusion strategy aims at harnessing the ability of DCs to present multiple tumor antigens to the host T cells^96^. Phase I studies demonstrated this vaccine to be safe and efficacious^97^. In a subsequent phase II study (*n* = 36), the administration of this vaccine in the postautologous stem cell transplant setting demonstrated a doubling of the myeloma-specific CD4+ and CD8+ T cells^98^. Seventy-eight percent patients achieved a ≥VGPR with this strategy. Notably, 24% patients who achieved only a PR postautologous transplant converted to a CR or near CR with the use of the fusion vaccine. However, loss of response over time seems to be present^98^. Combining IMiDs or checkpoint inhibitors with these vaccines may have some value in improving both the duration as well as amplitude of response to vaccine therapy^99,100^.

## Conclusions

Immune dysregulation plays a key role in the pathogenesis and disease progression of MM and restoration of a robust immune response toward the myeloma clone represents an important therapeutic strategy. The rapid evolution of CAR-T cell therapy and BiTEs has already started to reshape the treatment of RRMM.
